# Biological Properties and Antimicrobial Potential of Cocoa and Its Effects on Systemic and Oral Health

**DOI:** 10.3390/nu15183927

**Published:** 2023-09-10

**Authors:** Simone Ortiz Moura Fideles, Adriana de Cássia Ortiz, Carlos Henrique Bertoni Reis, Daniela Vieira Buchaim, Rogério Leone Buchaim

**Affiliations:** 1 Department of Biological Sciences, Bauru School of Dentistry (FOB/USP), University of Sao Paulo, Bauru 17012-901, Brazil; simoneortizf@gmail.com (S.O.M.F.); adrianacsortiz@gmail.com (A.d.C.O.); dr.carloshenriquereis@usp.br (C.H.B.R.); 2 Postgraduate Program in Structural and Functional Interactions in Rehabilitation, Postgraduate Department, University of Marilia (UNIMAR), Marília 17525-902, Brazil; danibuchaim@alumni.usp.br; 3 Medical School, University Center of Adamantina (UNIFAI), Adamantina 17800-000, Brazil; 4 Graduate Program in Anatomy of Domestic and Wild Animals, Faculty of Veterinary Medicine and Animal Science, University of São Paulo (FMVZ/USP), São Paulo 05508-270, Brazil

**Keywords:** cocoa, flavonoids, theobromine, antioxidants, antimicrobial, health benefits, systemic health, oral health

## Abstract

Cocoa is considered a functional food because it is a natural source of macro- and micronutrients. Thus, cocoa is rich in vitamins, minerals, fiber, fatty acids, methylxanthines and flavonoids. In addition to favoring the metabolism of lipids and carbohydrates, the bioactive components of cocoa can have an antioxidant, anti-inflammatory and antimicrobial effect, providing numerous benefits for health. This literature review presents an overview of the effects of cocoa, fruit of the *Theobroma cacao* tree, on systemic and oral health. Several studies report that cocoa intake may contribute to the prevention of cardiovascular, neurodegenerative, immunological, inflammatory, metabolic and bone diseases, in addition to reducing the risk of vascular alterations and cognitive dysfunctions. On oral health, in vitro studies have shown that cocoa extract exerted an inhibitory effect on the growth, adherence and metabolism of cariogenic and periodontopathogenic bacteria, also inhibiting acid production, glycosyltransferase enzyme activity and the synthesis of insoluble polysaccharides. Additionally, administration of cocoa extract reduced biofilm accumulation and caries development in animals infected with cariogenic species. Clinical studies also reported that the use of mouthwashes containing cocoa extract reduced *Streptococcus mutans* counts in saliva and dental biofilm formation. In short, these studies highlight the nutritional value of cocoa, considering its clinical applicability, stability and economic accessibility.

## 1. Introduction

Cocoa, derived from the *Theobroma cacao* tree, has been widely used by industry as an ingredient in various food products, like chocolate, jams and jellies [1]. In addition to the food industry, cocoa has also been used as a raw material for cosmetic or pharmaceutical formulations, such as oils, hydratants and cocoa butter products. The wide applicability and interest in the use of cacao is due to its nutritional value, biological properties, stability and economic accessibility. Considering the nutritional aspect, cocoa has important dietary properties, constituting a natural source of macronutrients, as proteins and lipids, and micronutrients, as vitamins and minerals [2,3]. These nutrients are essential for the regulation of metabolism and the maintenance of the biological activities of the organism.

The cocoa fruit is elongated and grooved, supported by a woody peduncle, and has a shell composed of three parts: epicarp, mesocarp and endocarp. The structure of the cocoa bean contains the following components: proteins (12.92 g), fats (48.15–60.5 g, carbohydrates (9.23 g) and fibers (4.9 g). The main fatty acids are oleic (31–35 g), stearic (33–35 g), palmitic (25–28 g) and linoleic (2–3.5 g). Therefore, cocoa has a variety of minerals, including phosphorus, magnesium, copper, potassium and calcium [1,2,3], whereas the composition of cocoa and consequently the content/profile of its bioactive compounds can vary according to the genetic characteristics of the species, geographical factors, environmental conditions, maturation stage, cultivation and processing methods. Minerals play a fundamental role in several biological processes and some of them act as enzymatic cofactors in the synthesis of macromolecules [1,2,3]. Cocoa is also a source of fiber [4] and methylxanthines, such as theobromine, caffeine and theophylline [3,4]. The cocoa husk is particularly rich in fiber, which favors intestinal function, improves the lipid profile and contributes to cardiovascular health [4]. Furthermore, cocoa is one of the most abundant foods in flavonoids, which are bioactive compounds that exhibit biological properties of great interest for health [2]. Among them, flavonoids have considerable antioxidant, anti-inflammatory, antiallergic, antitumor, antimicrobial and antiviral potential [5].

Flavonoids are phytochemicals present in citrus fruits, berries, vegetables, legumes, cocoa, grains and beverages, like coffee, green tea and red wine [6]. These phytochemicals belong to the class of polyphenols and are classified into several subgroups according to the chemical composition of the carbon chain [7]. The main subgroups of dietary flavonoids are flavanols, flavones, flavonols, flavanones, anthocyanidins and isoflavones [7]. In cocoa, the main flavonoids present in the bean and husk belong to the flavanols subgroup, as epicatechins, catechins, and procyanidins [8]. Thus, flavanols largely contribute to the antioxidant effect of cocoa [1,2,4,8]. Procyanidins, in turn, are responsible for the astringent taste of cocoa, due to complexation with saliva proteins. Other subgroups of flavonoids are also part of the cocoa composition, such as anthocyanins, flavonols (quercetin, isoquercitin), flavones (luteolin, vitexin), and flavanones (naringenin) [2]. Flavonoids derived from cocoa have the ability to form insoluble complexes by binding with carbohydrates and proteins, and they can interfere with the metabolism of these macromolecule [4,8].

Thus, the consumption of cocoa can improve health in several aspects. Both the beans and cocoa husk have biological properties and food applicability. Cocoa beans are used for the preparation of food products, including cocoa powder and chocolates [9]. The products obtained from the processing of the cocoa husk favor the metabolism of lipids and carbohydrates, as they contain considerable amounts of antioxidant polyphenols, in addition to being rich in soluble fiber [8]. In short, cocoa can be considered a functional food because it acts beneficially on health. Thus, this literature review aims to present an overview of the effects of cocoa on systemic and oral health.

## 2. Health Benefits of Cocoa

Several studies have reported that the consumption of cocoa can provide health benefits [2,3,4]. The internal and external structural part that forms the cocoa (husk, pulp, bean) are sources of macronutrients and micronutrients, such as carbohydrates, lipids, proteins, vitamins and mineral salts, which play a structural, functional and energetic biological role, as well as being rich in bioactive compounds, such as polyphenols and methylxanthines, which have potentially beneficial biological effects on health. The benefits from the use of cocoa are related to the biological properties of several active agents present in its composition, especially the flavonoids. The therapeutic action of flavonoids, in turn, is mainly attributed to their antioxidant and anti-inflammatory potential, considering that these properties are fundamental for the maintenance of tissue homeostasis [10,11,12,13].

Flavonoids act by eliminating reactive oxygen species (ROS) and minimizing the formation of ROS, as well as reducing the synthesis of important inflammatory mediators, such as tumor necrosis factor alpha (TNF-α), interleukin-6 (IL-6), interleukin-1 beta (IL-1β), cyclooxygenase-2 (COX- 2) and prostaglandin E2 (PGE2) [10,11]. Flavonoids also act by activating antioxidant enzymes and inhibiting enzymes involved with ROS production, as well as inhibiting the expression and synthesis of factors related to oxidative stress, as nitric oxide (NO) and nitric oxide synthase 2 inducible (iNOS) [12,13]. Neutralization and elimination of free radicals reduce toxicity and cellular oxidative damage, preventing deleterious changes in macromolecules, such as DNA [14]. Due to these properties, flavonoids and their metabolites may exert therapeutic potential, contributing to the prevention of cardiovascular, neurodegenerative, inflammatory and metabolic pathologies, like osteoarthritis and diabetes mellitus [7,10,15], in addition to sedentary and lifestyle-related diseases [14]. There is evidence in the literature that flavonoids may favor the reduction of plasmatic triglycerides and cholesterol levels and act by inhibiting tumor growth [16,17].

Flavonoids can also have a considerable neuroprotective effect, contributing to the preservation of nervous tissue. The antioxidant and anti-inflammatory potential of flavonoids contributes to this protective effect, considering that cellular oxidative stress and increased levels of inflammatory mediators alter tissue homeostasis, favoring nerve cell apoptosis, axonal degeneration and demyelination [18,19,20,21]. These molecular and tissue alterations, in turn, are frequently associated with the development of neurodegenerative diseases [19,20,22]. Flavonoids also act on bone cell metabolism, favoring the expression of factors that induce bone formation and inhibiting the expression of factors involved in matrix resorption [23,24,25,26]. The anti-inflammatory action of flavonoids contributes to minimize bone resorption by inhibiting the synthesis of inflammatory mediators [23,26]. The antioxidant and antiapoptotic potential of flavonoids, in turn, favor the survival of bone cells and contribute to the maintenance of tissue homeostasis [26]. Some flavonoids are even considered phytoestrogens, such as isoflavones. By stimulating osteogenesis, phytoestrogens exert an anabolic effect, contributing to the preservation of tissue structure and to the increase of bone mineral density [25].

In addition to flavonoids, cocoa has several other bioactive molecules (vitamins, mineral salts, methylxanthines, fibers, proteins and fatty acids) that, together, act beneficially on the different tissues of the organism. Studies report that bioactive agents in cocoa can act to reduce the risk of vascular and blood pressure alterations, coronary heart disease, stroke, cerebral oxidative stress, cognitive impairment and neurodegenerative disorders [2,4,14]. Thus, there are reports in the literature that cocoa may have a beneficial impact on cognitive functions [2,4], contributing to the prevention of neurodegenerative diseases and conditions resulting from aging [4]. A beneficial effect of these bioactive agents on inflammatory, immunological and metabolic diseases has also been suggested [2,14,27]. The bioactive compounds in cocoa, especially flavonoids and their metabolites, also exert an important prebiotic effect, favoring the growth of beneficial bacteria to the detriment of pathogenic ones [28]. This modulatory effect on the composition of the microbiota favors intestinal health and reduces the risk of various diseases [28].

With regard to bone, there is little evidence in the literature about the effects of cocoa consumption on this tissue. A clinical study conducted with women aged 70 to 85 years to investigate the effect of chocolate consumption on bone density and strength did not find a positive association between the frequency of chocolate consumption and an improvement in the evaluated bone parameters. This study considered the frequency of chocolate consumption, as rarely (<1 time/week), moderate (1–6 times/week) and daily (≥1 time/day). Daily consumption of chocolate (1 time/day), when compared to 1 time/week, showed a 3.1% lower body bone density [29]. Clough and colleagues (2017) showed that theobromine, one of the bioactive components of cocoa, is a xanthine alkaloid found in cocoa and cocoa-derived products, e.g., chocolate, and favors the osteogenic potential of bone marrow mesenchymal stem cells human in vitro [30]. In this study, the progeny of rats treated with theobromine during pregnancy and lactation, and that received theobromine for a period of time after weaning, showed greater body mass and more accelerated bone development. Microtomographic analysis of the trabecular structures of the tibia showed that the group treated with theobromine presented a considerable increase in the number of trabeculae and a reduction in the trabecular separation compared to the untreated group; however, between the groups, there was no expressive difference in the trabecular thickness. Taken together, the in vitro and in vivo data from this study pointed to a potential effect of theobromine on bone tissue [30].

The nutritional importance and beneficial effects of cocoa consumption on health were highlighted in a recent literature review [3]. This review compiled several studies that investigated the effects of cocoa on general health, considering different types of diseases or clinical conditions. This comprehensive study of the literature reported that there is a significant association between cocoa intake, in the form of chocolate, and reduced risk of coronary heart disease, estimating that this association can reduce the risk of developing cardiovascular disease and stroke in 37% and 29%, respectively. Some studies in this review reported that the consumption of products containing cocoa can have a vasoprotective effect, improving endothelial function. Other studies have observed a beneficial effect of flavonoids present in chocolate on blood pressure, especially in hypertensive individuals.

Few epidemiological studies have compared dark chocolate with milk chocolate. It is not clear what the ideal amount of chocolate is, given the lack of uniformity in dosing in randomized trials. Most studies performed do not specify the exact type or amount of chocolate used in individual studies. Generally, positive effects on cardiovascular health are seen with a higher percentage of cocoa. Clinical studies included in this review also reported that there was an improvement in cognitive functions by the use of cocoa or cocoa-derived products. Additionally, some studies have investigated the effects of cocoa consumption on cancer, but these data are not yet conclusive. This study also highlighted that research should advance to expand knowledge about the mechanisms of action and the biological effects of cocoa on health [3].

**Figure 1:**
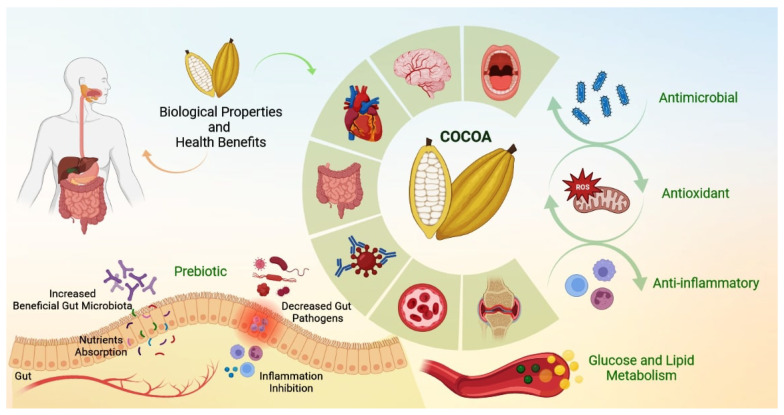
The bioactive components of cocoa have antioxidant, anti-inflammatory and antimicrobial potential. In addition to favoring the metabolism of carbohydrates and lipids, cocoa also exerts a prebiotic effect. Due to its properties, cocoa intake can contribute to the prevention of various disorders, such as cardiovascular, neurodegenerative, immunological, inflammatory, metabolic and bone diseases.

Figure 1 summarizes the main properties and biological effects of cocoa (pulp, cocoa beans and husk) on health.

## 3. Biological Properties of Cocoa and Oral Health

In addition to reducing the risk of various systemic diseases, the benefits of cocoa also extend to oral health. As with other organism tissues, the antioxidant and anti-inflammatory properties of cocoa can contribute to the maintenance of oral tissue homeostasis. Tomofugi et al. (2009) investigated the effect of cocoa administration on gingival oxidative stress in a model of ligature-induced periodontitis in rats [31]. In this study, serum levels of oxidative stress metabolites, levels of 8-hydroxydeoxyguanosine and reduced/oxidized glutathione ratio (GSH/GSSG) in gingival tissue were some of the main parameters used to assess the effect of treatment. Analyses performed after 4 weeks showed that animals with induced periodontitis and not treated with cocoa had significantly higher serum levels of TNF-α compared to animals in the control group (healthy animals) and animals with induced periodontitis that were treated with cocoa. Between the control and cocoa-treated groups, no significant differences in serum levels of TNF-α were detected. Additionally, significant histological alterations were observed in animals with periodontitis and not treated with cocoa, such as apical migration of the junctional epithelium, alveolar bone loss and infiltration of inflammatory cells. After 4 weeks, the degree of alveolar bone loss, polymorphonuclear leukocyte density, ratios of TNF-α-positive fibroblasts to total fibroblasts, and TRAP-positive osteoclasts were significantly higher in this group compared to the control and cocoa-treated groups. However, the animals treated with cocoa showed a significantly higher density of polymorphonuclear leukocytes than the control group. Thus, the GSH/GSSG ratio provides an insight into the redox status of cells and the data from this study showed significant differences with respect to this parameter. The authors [31] describe that the gingival levels of 8-hydroxydeoxyguanosine in the cocoa-treated group showed a 25% decrease compared to the group of animals with periodontitis and not treated with cocoa, but the levels of this factor in the cocoa-treated group were 60% higher than the control. Thus, GSH/GSSG ratio of the cocoa-treated group increased by 58% in relation to the group of animals with periodontitis and not treated with cocoa, but this rate was 16% lower than the control. Taken together, these data evidenced that cocoa exerted a beneficial effect on antioxidant and anti-inflammatory status in animals with induced periodontitis. In addition to these analyses, the evaluation of the levels of 8-hydroxydeoxyguanosine and reduced/oxidized glutathione ratio (GSH/GSSG) in the gingival tissue also showed relevant data, considering that 8-hydroxydeoxyguanosine constitutes a biomarker of oxidative damage to DNA [32] and glutathione acts as an antioxidant agent [33].

In addition to their antioxidant and anti-inflammatory properties, studies have reported that flavonoids may exert an anticollagenolytic effect. Considering that flavonoids constitute one of the main bioactive components of cocoa, a beneficial action on dental tissue could be expected by the use of these agents. According to literature data, flavonoids may contribute to the maintenance of dental tissue integrity by inhibiting the expression and activity of matrix metalloproteinases (MMPs) [15,16,34,35,36]. MMPs are endopeptidases responsible for the degradation of proteins, including dentin collagen [36]. In the oral environment, MMPs present in dentin can be activated by enzymes or bacterial products from caries and dental biofilm activity [37,38]. Thus, the activity of MMPs contributes to the degradation of the dentin organic matrix, favoring the loss of dental tissue [37,38]. The biological action of flavonoids on the dentin matrix has been reported in several studies. In vitro studies showed that flavonoids inhibited the synthesis of PGE2 and several types of MMPs, as well as the proteolytic activity of these enzymes [15,34,35]. Other studies have shown that treatment of specimens with flavonoids stabilized collagen fibres, preserving the dentin matrix, even in deep lesions not exposed to fluoride [39,40]. Corroborating these data, an in situ study found that the treatment of specimens with flavonoid minimized degradation of the dentin matrix [36]. A later study showed that treatment of demineralized dentin with flavonoids preserved collagen fibers and dentin matrix in a dose-dependent manner [35]. These studies showed that flavonoids act by stabilizing collagen fibers and inhibiting the activity of proteolytic enzymes, which may contribute to the preservation of dental tissue [15,35,36]. Thus, the use of agents with anticollagenolytic potential could have great applicability in the oral environment.

Besides these properties, there is also evidence in the literature that the bioactive components of cocoa may favor oral health by exerting antibacterial effect on cariogenic and periodontopathogenic species.

### Antimicrobial Potential of Cocoa

The antimicrobial potential of cocoa (husk and cocoa bean extract) has been reported in several studies. The effects of cocoa husk extract or cocoa beans on oral microorganisms, mainly against cariogenic bacteria, have been investigated by several in vitro studies. In 1979, Palenik and colleagues had already observed that components of cocoa bean husk extract inhibited the adherence and growth of *S. mutans* in vitro, as well as the synthesis of insoluble extracellular polysaccharides [41]. Later, Osawa et al. (2001) found that cocoa bean husk extract exhibited inhibitory activity on the enzyme glycosyltransferase and antibacterial effect on *S. mutans* cultures [42]. In this study, through chromatographic analyses, several bioactive compounds present in the cocoa bean husk were identified, which can be related to the anti-glycosyltransferase activity, as polyphenols (epicatechins), and with the antibacterial effect on *S. mutans*, like fatty acids, especially oleic and linoleic acids [42]. Subsequent studies that obtained similar results showed that cocoa bean husk extract reduced the viability and adherence of *S. mutans* in vitro, as well as acid production and insoluble polysaccharide synthesis in bacterial cultures [43,44,45]. Polyphenols extracted from cocoa beans also reduced biofilm formation by *S. mutans* and *S. sanguinis* in vitro, as well as acid production by *S. mutans* [46]. Additionally, Matsumoto et al. (2004) found a significant reduction in the number of viable *S. mutans* cells in human biofilms collected from children aged 4 to 15 years, after in vitro exposure of this biofilm to cocoa bean husk extract for 1 h [43].

The study by Lagha et al. (2021) investigated the effect of cocoa extract on adherence, growth and biofilm formation by *Fusobacterium nucleatum*, a microorganism involved in the development and progression of periodontal disease, among other pathologies [47]. This in vitro study showed that cocoa extract reduced the growth and biofilm formation, and inhibited the adherence of *F. nucleatum* to oral epithelial cells, in a dose-dependent manner. In addition to the antimicrobial effect, the cocoa extract showed an anti-inflammatory effect, reducing the secretion of cytokines IL-6 and interleukin-8 (IL-8) by oral epithelial cells stimulated by *F. Nucleatum*. These data showed that the cocoa extract disturbed the virulence of this pathogen, showing the potential to act as a supporting agent in the prevention and treatment of periodontal disease [47].

There is also evidence in the literature that theobromine may have antimicrobial potential against oral species. Lakshmi et al. (2019) evaluated the in vitro effect of fluoridated and nonfluoridated children’s dentifrices containing theobromine against some strains of microorganisms, including *S. mutans*, *Lactobacillus acidophilus* and *Enterococcus faecalis* [48]. This study reported that dentifrice containing theobromine showed the largest zones of inhibition and consequently higher antibacterial activity compared to fluoridated dentifrices [48]. Demir et al. (2021) investigated the antimicrobial potential of dentifrices containing traditional ingredients and of various dentifrices containing different natural active agents (theobromine, aloe vera, miswak, propolis, chitosan, enzymes and probiotics) on cultures of oral bacteria, specifically *S. mutans*, *S. sanguinis* and *Enterococcus faecalis* [49]. The effect of dentifrices on bacterial cultures was measured by the diameters of the inhibition zones after 24 h of incubation. The results showed that the positive control (mouthwash containing 0.2% chlorhexidine digluconate) used in the study presented, in relation to the dentifrices, a significantly greater antimicrobial efficacy against all the tested bacteria. Among the tested dentifrices, the dentifrice containing traditional ingredients showed the highest antimicrobial efficacy against *S. mutans*, followed by the dentifrices containing theobromine, miswak, chitosan, aloe vera and propolis. However, among the natural agents, theobromine presented the best antimicrobial effect against *S. mutans*, not differing significantly from the traditional dentifrice. The study also reported that only toothpastes containing traditional ingredients, theobromine and chitosan had an antimicrobial effect against *S. sanguinis* and *E. faecalis*, with the traditional toothpaste having the best effect, differing significantly from the others [49]. Thus, considering the increasing interest in natural products, the use of antimicrobial products containing bioactive agents may constitute a promising option, although research has yet to advance in this regard.

**Figure 2:**
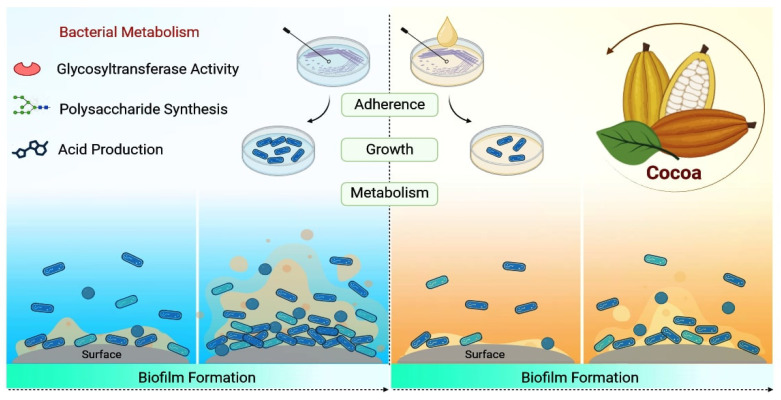
Cocoa extracts exert an inhibitory effect on the adherence, growth and metabolism of oral bacteria, disturbing biofilm formation. The bioactive components of cocoa inhibit acid production, glycosyltransferase enzyme activity and the synthesis of insoluble polysaccharides.

Figure 2 schematizes the inhibitory effects of cocoa on bacterial growth and biofilm formation in vitro.

The effect of cocoa on oral microorganisms was further demonstrated in a study conducted with animal models. Ooshima et al. (2000) found that cocoa bean husk extract could have considerable anticariogenic potential in vivo [44]. This study investigated the effect of cocoa bean husk extract on caries development and dental biofilm accumulation in rats infected with *S. mutans* or *S. sobrinus*. These animals were treated with sucrose and, at weekly intervals, oral swabs were collected for microbiological analysis. The results of the analyses showed that cocoa bean husk extract significantly reduced biofilm accumulation and caries development in rats infected with *S. mutans* or *S. sobrinus*. In both experiments, reductions in plaque index and caries scores were obtained by using cocoa bean husk extract at concentrations higher than 0.5 mg/mL and 1.0 mg/mL, respectively [44]. In addition, a recent study that investigated the antimicrobial effect of several extracts obtained from a variety of cocoa beans from different origins reported that almost all of the tested extracts showed activity against *S. mutans* [50].

Corroborating the data from in vitro studies and animal models, clinical studies that investigated the antimicrobial potential of cocoa obtained promising results. Several clinical studies have shown that the use of mouthwashes containing cocoa extract significantly reduced the count of *S. mutans* in saliva and the formation of dental biofilm [43,51,52,53,54,55]. A study performed with 28 individuals (19–29 years) who used a mouthwash containing cocoa bean husk extract for 4 days obtained satisfactory results in relation to the use of a mouthwash containing only the vehicle (control) [43]. In this study, there was a significant reduction in the accumulation of dental biofilm and in the number of *S. mutans* in saliva, even in the absence of oral hygiene procedures. However, no significant changes were observed in the number of total streptococci in saliva, measured by colony-forming units (CFU). Additionally, no adverse or undesirable effects were reported from the use of mouthwash containing cocoa extract [43]. Srikanth et al. (2019) also showed that the use of mouthwash containing cocoa can have considerable antibacterial effect [51]. In this crossover study, 32 children (10–14 years) used, after dental prophylaxis, a mouthwash containing 0.1% cocoa bean husk extract or a placebo mouthwash (control) for 4 days, without any oral hygiene procedure during this period. Microbiological analysis was performed on samples of unstimulated saliva collected for colony-forming unit counting. Additionally, the plaque index was evaluated using the modified Quigley and Hein Index. The results showed that the use of mouthwash containing cocoa bean husk extract reduced *S. mutans* count and plaque index scores by 20.9% and 49.6%, respectively, differing statistically from the control [51]. A study conducted with 30 children (12–14 years) also obtained a significant reduction in the number of *S. mutans*, measured in colony-forming units (CFU) in saliva collected 30 min after the use of a mouthwash containing 0.1% cocoa extract [52]. According to this study, flavanols, as epicatechins, and fatty acids, as oleic acid, may have contributed considerably to the antibacterial action of cocoa extract [52].

Clinical studies that compared the effect of mouthwashes containing chlorhexidine or cocoa extract showed a significant reduction of *S. mutans* in the saliva of both experimental groups, suggesting that cocoa may present an antimicrobial effect similar to chlorhexidine [53,54]. According to these studies, cocoa may represent a low-cost therapeutic alternative for use as an antimicrobial agent [53,54]. Babu et al. (2011) conducted a study with 50 children aged 6 to 10 years to evaluate the antimicrobial effect of mouthwash containing 0.1% cocoa bean husk extract or mouthwash containing 0.2% chlorhexidine [53]. In this study, saliva samples were collected at different periods (baseline, 7 days, 1 and 2 months) for *S. mutans* count analysis. The results showed a significant reduction in the count of *S. mutans* for the groups that used mouthwashes containing chlorhexidine (23.2%) or cocoa bean husk extract (22.4%), with no significant difference between them. According to the study data, cocoa may be an interesting option as an antimicrobial agent for children [53]. Shrimathi et al. (2019) conducted a randomized controlled crossover study that investigated the antibacterial potential of mouthwashes containing cocoa bean husk extract (0.5%), chlorhexidine (0.2%) or ginger (12.5%) [54]. In this study, 75 individuals (18 to 25 years) used the mouthwashes for 7 days. Analysis of saliva samples showed that the mouthwashes containing cocoa extract or chlorhexidine significantly reduced the population of *S. mutans*, while the mouthwash containing ginger resulted in a significant reduction in the number of *Lactobacillus*. This study highlighted that mouthwashes containing natural products may constitute an economical and effective strategy for use as oral antimicrobials [54]. A recent study conducted by Kibriya et al. (2023) also showed that cocoa bean husk extract may have considerable activity against cariogenic bacteria [55]. This research aimed to evaluate the antibacterial effect of mouthwashes containing 0.1% cocoa bean husk extract, used with different vehicles (distilled water, Ringer’s lactate or saline solution), 0.12% chlorhexidine or 0.05% sodium fluoride (NaF). In this study, 80 children aged 7 to 12 years used the mouthwashes twice a day for a period of up to 14 days. The effect of the treatments was evaluated mainly by measuring the plaque index (Simplified Oral Hygiene Index) and by counting of *S. mutans* colony-forming units in unstimulated saliva, at different experimental periods (baseline, 1, 7 and 14 days). The results showed that the lowest plaque index scores were presented by groups treated with mouthwash containing cocoa bean husk extract, in distilled water or Ringer’s lactate, or mouthwash containing chlorhexidine. The highest plaque index scores were found in the group treated with mouthwash containing NaF. Regarding the count of the number of *S. mutans* in saliva (CFU/mL), the study reports that the mouthwash containing cocoa bean husk extract in saline solution showed the best antibacterial effect in relation to the other groups, in practically all periods evaluated. Considering the data obtained, this study concluded that the mouthwash containing cocoa extract can act as an effective antibacterial agent to control caries and gingivitis, constituting a safe alternative for use by children [55].

Table 1 summarizes the main outcomes of clinical studies that investigated the antimicrobial potential of mouthwashes containing cocoa extract.

Studies in the literature have evaluated the antimicrobial effects of cocoa on children (Table 1). According to these studies, evaluating the use of an oral hygiene product that has a potential antimicrobial effect and that constitutes a safe alternative for use by children was the main objective of these investigations. Thus, these studies were relevant to investigate a compound/product that has therapeutic potential in the control and prevention of dental caries, which still causes a great impact on children’s oral health and extends into adulthood.

**Figure 3:**
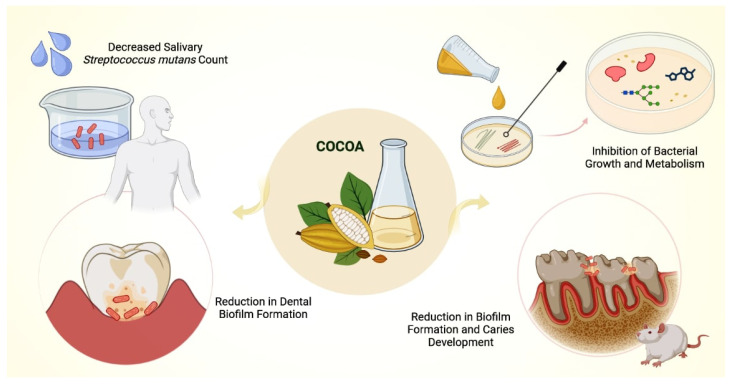
This illustration presents an overview of the main antimicrobial effects of cocoa on oral bacteria. Cocoa extract inhibited bacterial adherence, growth and metabolism in vitro, reduced biofilm formation and caries development in animal models, and reduced salivary *S. mutans* counts and plaque index scores in clinical trials.

Figure 3 provides an overview of the main outcomes from in vitro, animal models and clinical studies that investigated the effects of cocoa on oral microorganisms.

## 4. Conclusions and Future Perspectives

Cocoa is rich in nutrients with antioxidant and anti-inflammatory properties, being considered a functional food. Studies have shown that the bioactive agents present in the composition of cocoa, especially flavonoids, can act to reduce the risk of intestinal, cardiovascular, metabolic, neurological, inflammatory and immunological disorders, among others. However, although there is evidence of the benefits that the ingestion of products containing cocoa can provide to health, more research is needed to dimension its biological activity considering the different physiopathological conditions of the organism, as well as to elucidate the data that are still controversial. With regard to oral health, additional studies are also needed to expand the investigations on the antimicrobial potential of cocoa components, considering the diversity of bacterial species involved in the formation of dental biofilm and its relationship with the caries process. In addition to the variability in the dietary patterns of each population, as well as in the experimental protocols of each study, it is important to emphasize that differences related to cocoa, such as genetic diversity and variety of beans, geographical distribution and forms of cultivation, should also be considered in investigative analyses of their biological properties. However, regardless of these particularities, cocoa has been highlighted as a natural source of polyphenols and bioactives of great interest for clinical and dietary application.

## Figures and Tables

**Table 1:** Clinical studies that investigated the antimicrobial potential of mouthwashes containing cocoa extract.

References	Participants	Intervention	Main Outcomes
Babu et al. (2011) [53]	50 children (6–10 years)	Use of mouthwash containing 0.1% cocoa bean husk extract or mouthwash containing 0.2% chlorhexidine for up to 2 months.	Mouthwashes containing 0.2% chlorhexidine (23.2%) or 0.1% cocoa bean husk extract (22.4%) significantly reduced *S. mutans* counts (CFU) in saliva, with no statistical differences between them.
Matsumoto et al. (2014) [43]	28 participants (19–29 years)	Use of mouthwash containing cocoa bean husk extract or vehicle for 4 days, without oral hygiene procedures.	Mouthwash containing cocoa bean husk extract significantly reduced dental biofilm accumulation and *S. mutans* counts in saliva, however, without expressive alterations in the total count of salivary streptococci (CFU).No adverse or undesirable effects were reported.
Fajriani et al. (2016) [52]	30 children (12–14 years)	A single rinse with mouthwash containing 0.1% cocoa extract.	Analysis of saliva collected 15 and 30 min after rinsing showed that cocoa extract significantly reduced the *S. mutans* count (CFU) in relation to baseline.
Srikanth et al. (2019) [51]	32 children (10–14 years)	Use of mouthwash containing 0.1% cocoa bean husk extract or a placebo mouthwash (control) for 4 days, without oral hygiene procedures.	Mouthwash containing 0.1% cocoa bean husk extract reduced *S. mutans* count (CFU) and plaque index scores (modified Quigley and Hein Index) by 20.9% and 49.6%, respectively, differing statistically from the control.
Shrimathi et al. (2019) [54]	75 participants (18–25 years)	Use of mouthwashes containing cocoa bean husk extract (0.5%), chlorhexidine (0.2%) or ginger (12.5%) for 7 days.	Mouthwashes containing cocoa extract or chlorhexidine significantly reduced the population of *S. mutans*, while the mouthwash containing ginger resulted in a significant reduction in *Lactobacillus* levels (CFU).
Kibriya et al. (2023) [55]	80 children (7–12 years)	Use of mouthwashes containing 0.1% cocoa bean husk extract (tested with different vehicles, such as distilled water, Ringer’s lactate or saline solution), 0.12% chlorhexidine or 0.05% sodium fluoride (NaF) for up to 14 days.	The groups treated with mouthwash containing cocoa bean husk extract, in distilled water or Ringer’s lactate, or mouthwash containing chlorhexidine showed the lowest plaque index scores (Simplified Oral Hygiene Index). Considering the *S. mutans* count (CFU) in saliva, the mouthwash containing cocoa husk extract in saline solution showed the best antibacterial effect.

CFU: Colony-Forming Units.

## Data Availability

Not applicable.
